# Propolis potentiates the effect of cranberry (*Vaccinium macrocarpon*) in reducing the motility and the biofilm formation of uropathogenic *Escherichia coli*

**DOI:** 10.1371/journal.pone.0202609

**Published:** 2018-08-23

**Authors:** Jérémy Ranfaing, Catherine Dunyach-Remy, Jean-Philippe Lavigne, Albert Sotto

**Affiliations:** 1 French National Institute of Health and Medical Research Unit 1047, University Montpellier, Faculty of Medicine, Nîmes, France; 2 Department of Microbiology, Nîmes University Hospital, Nîmes, France; 3 Department of Infectious Diseases, Nîmes University Hospital, Nîmes, France; RMIT University, School of Science, AUSTRALIA

## Abstract

One strategy to prevent urinary tract infections is the use of natural products such as cranberry (*Vaccinium macrocarpon*) and propolis. The objective of this study was to evaluate the impact of these products alone and combined on the motility and biofilm formation of a collection of representative uropathogenic *Escherichia coli* (UPEC). Motility was evaluated by the swarming and swimming capacity of the isolates in presence/absence of cranberry ± propolis. Early and late biofilm formation was observed with the Biofilm Ring test (BioFilm Control) and the crystal violet method. Cranberry alone was seen to have a variable effect on motility and biofilm formation unrelated to bacterial characteristics, but a reduced motility and biofilm formation was observed for all the isolates in the presence of cranberry + propolis. These results suggest that cranberry alone doesn’t work on all the *E*. *coli* strains and propolis potentiates the effect of cranberry on UPEC, representing a new strategy to prevent recurrent urinary tract infections.

## Introduction

Urinary Tract Infections (UTIs) are the most common bacterial infections in both community and hospital settings [1]. It has been estimated that about 150 million people worldwide develop UTI each year, with high costs in terms of hospitalizations and medical expenses [2–4]. Among the common uropathogens associated with UTIs development, Uropathogenic *Escherichia coli* (UPEC) is the primary cause [5]. Effective methods to prevent UTI have been sought for many years, particularly since the beginning of the century due to the diffusion of multidrug resistance *E*. *coli* [6–9].

UPEC strains possess a plethora of both structural (e.g., fimbriae, pili, curli, flagella) and secreted (toxins, iron-acquisition systems) virulence factors that contribute to their pathogenicity. The ability to adhere to host epithelial cells in the urinary tract represents the most important determinant of pathogenicity [10]. Different strategies (e.g., vaccines, natural antimicrobial compounds, anti-adhesive peptides) have been proposed targeting these virulence factors [10]. Two natural substances were identified (cranberry (*Vaccinium macrocarpon* Ait.) and propolis) effective to prevent UTIs [7, 11–15]. The antimicrobial effect of cranberry is largely due to the A-type proanthocyanidins (PAC-A), the dominating compound found in cranberries. PAC-As impair bacterial adherence by inhibiting Type-I fimbriae UPEC adhesion to uroepithelial cells [16]. They also trigger cell rounding, thus reducing its surface of adherence [17]. PAC-As reduce the mobility of UPEC, a crucial step in the establishment of the ascending infection (passage between the ureter to the bladder and the bladder to the kidney), as seen in *E*. *coli* [14] and in *Proteus mirabilis* [18]. Finally, cranberry modifies biofilm formation, another important metabolic pathway involved in recurrent UTI and catheter-associated UTI (CAUTI) [19], as has been observed in *Enterococcus faecalis* [20], *E*. *coli* [21] and *Pseudomonas aeruginosa* [22]. Propolis, a resinous material collected by bees from plants, has antimicrobial, anti-inflammatory, anti-tumour, immuno-modulatory and anti-oxidant activities [23]. Combined with cranberry, this substance can impact UPEC anti-adhesion activity, bacterial multiplication and virulence [16, 24–26]. The Cochrane Database concluded that no significant benefit was demonstrated for probiotics to prevent UTI [2].

Whilst the effect of cranberry on adhesion of UPEC has been extensively studied [6,7,12,16,21,26,27], the data on motility and biofilm formation are scarce. The objective of this study was to evaluate the impact of cranberry, propolis and a combination of these components on motility and biofilm formation of a collection of UPEC strains.

## Material and methods

### Bacterial strain, microbial culture and preparation of extracts

All the assays were performed with a collection of 12 UPEC strains previously isolated from patients with cystitis, pyelonephritis or asymptomatic UTI (colonisation) [28] (Table 1).

**Table 1:** Characteristics of the clinical Uropathogenic *Escherichia coli* strains used in this study [28].

Strains	Clinical aspects	Resistance profile^a^	ß-lactam content	Phylogroup	Main virulence factors
G03	Pyelonephritis	AMX, TIC, NAL, OFX, SXT, FUR	TEM-1	D	*papG2*, *papA*, *papE*, *fimH*, *iroN*, *kpsM II*, *k2 kps*, *iutA*, *traT*, *malX*, *irp2*, *cnf1*
G06	Pyelonephritis	AMX, AMC, TIC, TCC, TZP, CAZ, CTX, CXM, NAL, OFX, SXT	TEM-24	A	*papG2*, *fimH*, *iroN*, *kpsM II*, *iutA*, *traT*, *malX*
G08	Cystitis	AMX, TIC	TEM-1	A	*fimH*, *iroN*, *malX*, *irp2*
G10	Colonisation	-	-	A	*fimH*, *iroN*, *traT*, *malX*
G13	Cystitis	-	-	A	*papA*, *papC*, *fimH*, *iroN*, *kpsM II*, *k2 kps*, *iutA*, *traT*, *malX*, *irp2*
G19	Pyelonephritis	AMX, TIC, NAL, OFX	TEM-1	B1	*fimH*, *iroN*, *iutA*, *traT*, *malX*, *irp2*
G24	Colonisation	-	-	B1	*fimH*, *traT*, *malX*
G29	Colonisation	-	-	D	*fimH*, *iroN*, *kpsM II*, *k2 kps*, *iutA*, *traT*,
G39	Colonisation	AMX, AMC, TIC, TCC, TZP, CAZ, CTX, CXM, FEP, NAL, OFX, CIP, FUR	CTX-M-15, TEM-1	B2	*fimH*, *kpsM II*, *iutA*, *traT*, *malX*, *irp2*
G43	Colonisation	AMX, AMC, TIC, TCC CXM, SXT	TEM-1	B2	*papG2*, *papA*, *papC*, *papE*, *fimH*, *hlyB*, *iroN*, *kpsM II*, *k2 kps*, *iutA*, *traT*, *Irp2*, *cnf1*
G46	Cystitis	-	-	B2	*papG2*, *papA*, *papC*, *papE*, *fimH*, *iroN*, *kpsM II*, *k2 kps*, *iutA*, *traT*, *malX*, *irp2*,
G50	Cystitis	AMX, TIC, SXT	TEM-1	B2	*papG3*, *papA*, *papC*, *papE*, *fimH*, *hlyB*, *iroN*, *kpsM II*, *k2 kps*, *iutA*, *traT irp2*, *cnf1*, *sfa*

^a^AMX, amoxicillin; AMC, amoxicillin+clavulanic acid; TIC, ticarcillin; TCC, ticarcillin+clavulanic acid; TZP, tazocillin; CAZ, ceftazidime; CTX, cefotaxime; CXM, cefixime; NAL, nalidixic acid; OFX, ofloxacin; CIP, ciprofloxacin; SXT, cotrimoxazole; FUR, furadantin

A mix of filtered urine (filter by a vacuum-driven filtration system with a 0.22μm membrane, Millipore (Billerica, Massachusetts, USA)) and Luria Bertani (LB) broth growth medium (Invitrogen, Villebon sur Yvette) have been used for the overnight culture of our strains.

A Brain-Heart Infusion (BHI) growth medium (CondaLab, Madrid, Spain) has been used for Biofilm Ring-Test and crystal violet experiments. A LB growth medium culture has been used for mobility assays.

Cranberry extract (*V*. *macrocarpon*) was obtained by using dried cranberry (Exocyan cran BL-DMAC 6% (Nexira, Rouen, France)) and phosphate buffered saline (PBS) and sterilized by filtration. Purified PAC extract was prepared from fresh cranberry fruit by reverse phase and adsorption chromatography, as previously described [12,29] and stored under nitrogen at 4°C to prevent oxidation The concentration of PAC-A was measured by BL-DMAC (colorimetric method) [30] and liquid chromatography [31]. Final concentration of PAC-A used was standardized to contain 190 μg/L. Cranberry juice was stored at -20°C in the dark. The propolis extract (Plantex, Sainte-Geneviève-des-Bois, France) used in this study is a hydroalcoholic extract of blended propolis. The product was characterized by HPLC showing 2% of galangin (Lot 38123). The propolis was diluted in 50 mL of PBS and incubated at 37°C by shaking at 100 rpm for 8 hours. Then, the solution was clarified by centrifugation (4000 rpm, 20°C, 10 min). Supernatant was sterilized by filtration.

### Motility assays

Two forms of motility were studied: swimming and swarming. Swimming is used by the bacteria to move in a liquid medium and swarming in a semi-liquid medium. The motility of studied strains in different conditions was evaluated using soft agar LB plates as described previously [32]: swim plates containing 0.25% of agar and swarm plates containing 0.5% of agar supplemented with 0.5% of glucose. Briefly, bacteria grown overnight in LB were diluted 1000-fold in LB and incubated at 37°C to an OD600 of ≈ 0.7. Swarm plates were inoculated into the middle of soft agar by spotting with 5 μL of standardized culture. Swimming plates were seeded with the same inoculum below the agar surface using a sterile inoculating needle. Plates were incubated for 24 and 48 h at 37°C. The diameter of the migration zones produced by the strain at different conditions was calculated using Image J software. Swimming and swarming experiments were performed independently three times.

### Kinetics of biofilm formation

Kinetics of early biofilm was explored using the Biofilm Ring Test (BioFilm Control, Saint Beauzire, France) as described [33]. Briefly, standardized bacterial culture were incubated at 37°C in 96-well microtiter plates in the presence of magnetic beads. After different time points (0, 2 and 5 hours), plates were placed onto a magnetic block during 1 min then in the reader (Epson scanner modified for microplate reading). The images of each well before and after magnetic attraction were analysed with the BioFilm Control software giving a BioFilm Index (BFI). A high BFI value (> 7) indicates a high mobility of beads under magnet action (corresponding to an absence of biofilm formation) while a low value (< 2) corresponds to a complete immobilization of beads due to the sessile cells. Two independent experiments with at least two repeats were performed per conditions tested (cranberry with/without propolis) and per incubation time.

### Measure of constituted biofilm by crystal violet

Biofilm development was also assessed by incubating bacterial cultures, after an overnight incubation at 37°C and a dilution to obtain a final optical density at OD600 to ≈1, in 96-well microtiter plates in BHI. The plates were incubated at 37°C for 48 hours. After incubation, adherent cells were fixed with methanol (99%) and after different washing steps with distilled water, the crystal violet (0.1%) was added for 10 min at room temperature. The biofilm was then dissolved with acetic acid (33%). At least the liquid was read at OD620 [22].

### Statistical analysis

Statistics and graphs were prepared using the software package GraphPad Prism 6.0. The effects of cranberry and/or propolis on motility were assessed using one-way ANOVA followed by Dunnett's multiple comparisons test. The comparisons were estimated between untreated and treated strains. Kinetics of biofilm formation were compared with a two-way ANOVA followed by Dunnett's multiple comparisons test. The crystal violet experiments were assessed using a Student’s t-test. p < 0.05 was considered to reflect a statistically significant difference.

## Results

### Panel of studied strains

The 12 strains were representative of the most commonly isolates found in UTI.

For resistance, the panel contains 5 strains susceptible to all the main antibiotic used in UTI (G10, G13, G24, G29, G46), 2 strains harbouring a penicillinase (G8 and G50), 2 strains harbouring a penicillinase and a chromosome-mediated resistance to fluoroquinolones (G03 and G19), 1 strain harbouring a penicillinase and an overexpression of efflux pump (G43) and finally 2 main Extended-spectrum ß-lactamases (ESBL)-producing strains (G6 and G19), G6 belonging to the worldwide O25b-ST131-B2 clone [8].

For virulence traits, the half of strains belongs to B2 and D phylogroups related to the virulent UPEC and the other strains belong to the commensal *E*. *coli* (phylogroups A and B1). All the isolates harbour *fimH* gene which encodes the main pili of UPEC strains. Genes encoding other fimbriae (*papG2*, *papG3*) are differently distributed in the isolates. In the same way the genes involved in hemolysin (*hlyA*), capsule synthesis (*kpsM II*, *k2 kps*), iron acquisition (*iroN*, *irp2*), and cytotoxicity (*cnf1*) have a variable distribution in correlation with the main UPEC strains found in UTI [28].

### Treatment with cranberry + propolis inhibits UPEC motility

**Fig 1:**
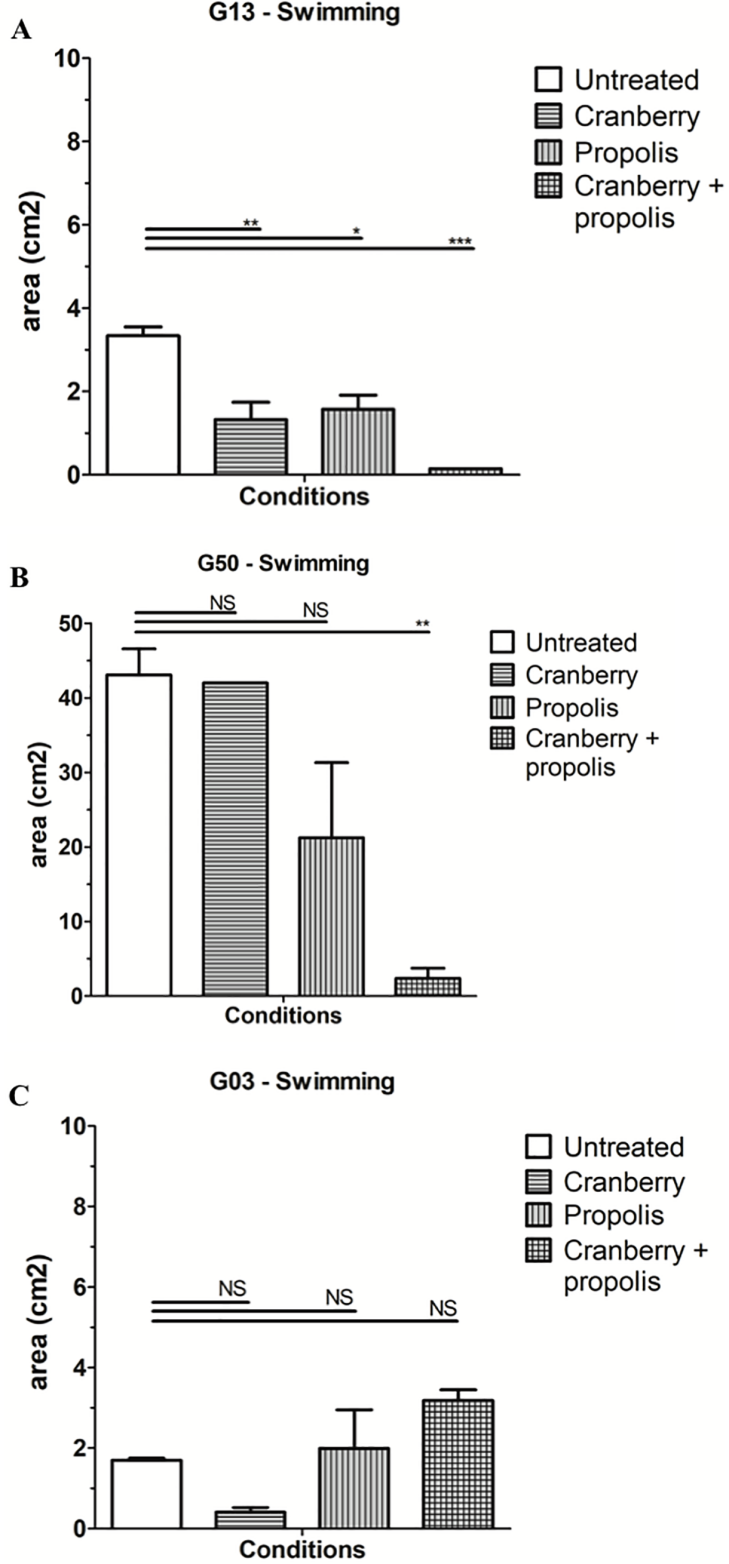
Representation of 3 different profiles observed on swimming capacity of a panel of uropathogenic *Escherichia coli* in presence of cranberry, propolis and both. Comparative results of swimming assays for representative strains in different growing conditions (cranberry, propolis, and both) at 48 hours. A) Profile of the swimming of the G13 strain B) Profile of the swimming of the G50 strain C) Profile of the swimming of the G03 strain The errors bars represent the standard deviation from at least two independent assays. *, p<0.01; **, p<0.001; ***, p<0.0001; NS, not significant.

To confirm the impact of cranberry ± propolis on bacterial mobility, swimming and swarming motilities were quantified on soft agar at 48 hours. Strains could be split into three different groups based on their motility without treatment and their response to the treatment, independent of virulence and resistance profiles (Fig 1).

First, eight strains (G08, G10, G13, G19, G24, G29, G39 and G46) show a moderate swimming without treatment and impaired swimming upon exposure to cranberry, propolis and the combination of both (mean swimming diameter of untreated *E*. *coli*: 3.34 cm^2^ ± 0.29; cranberry treatment: 1.32 cm^2^ ± 0.58 (p<0.0001); propolis treatment: 1.57 cm^2^ ± 0.47 (p<0.0001); cranberry + propolis treatment: 0.15 cm^2^ ± 0 (p<0.0001)) (Fig 1A). Two strains (G06 and G50) are a high swimming without treatment and are impacted only by the treatment with cranberry + propolis (mean swimming diameter of untreated: 43.07 cm^2^ ± 6.02; vs. cranberry treatment: 42.0 cm^2^ ± 0 (p = Non significant (NS); propolis treatment: 21.23 cm^2^ ± 17.45 (p = NS); cranberry + propolis treatment: 2.36 cm^2^ ± 1.93 (p<0.0001)) (Fig 1B). Finally, two strains (G03 and G43) are no motility and are not impacted by any of the treatments (mean swimming diameter of untreated: 1.7 cm^2^ ± 0.07; cranberry treatment: 0.41 cm^2^ ± 0.15 (p = NS); propolis treatment: 1.99 cm^2^ ± 1.35 (p = NS); cranberry + propolis treatment: 3.18 cm^2^ ± 0.37 (p = NS)) (Fig 1C).

**Fig 2:**
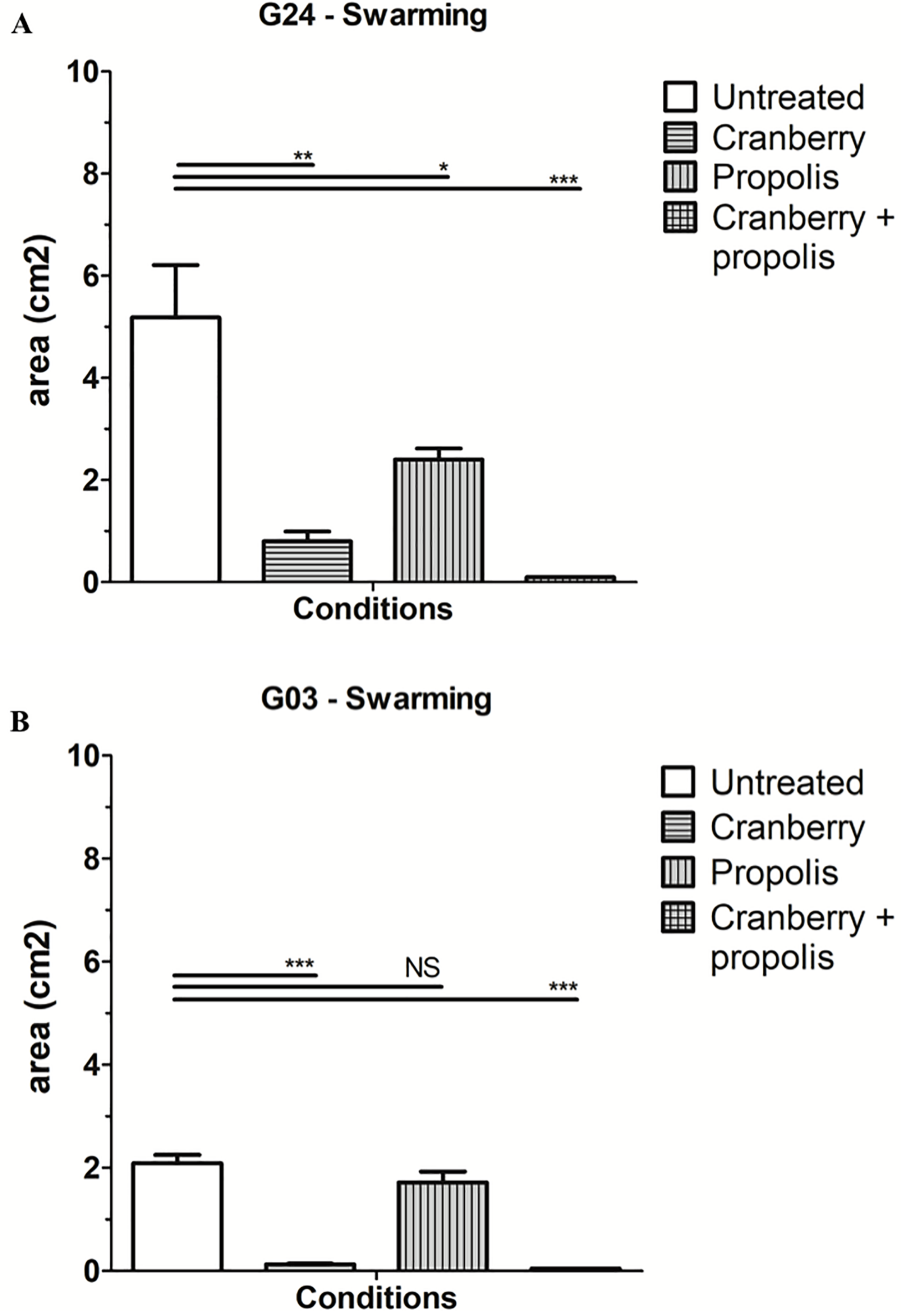
Effect of cranberry, propolis and both on swarming capacity of uropathogenic *Escherichia coli*. Comparative results of swarming assays for a representative strain (G24) in different growing conditions (cranberry, propolis, and combination of both) at 48 hours. A) Profile of the swarming of the G24 strain B) Profile of the swarming of the G03 strain The errors bars represent the standard deviation from at least two independent assays.

A significant impact of cranberry alone and cranberry + propolis treatment on swarming was also observed for all strains (Fig 2). As a representative profile, strain G24 shows a mean swarming diameter untreated: 5.19 cm^2^ ± 1.77; cranberry treatment: 0.8 cm^2^ ± 0.26 (p<0.0001); propolis treatment: 2.1 cm2 ± 0.25 (p<0.05); cranberry + propolis: 0.1 cm^2^ ± 0 (p<0.0001)) (Fig 2A). For five strains (G03, G10, G29, G39 and G50), the difference between the untreated condition and the propolis condition was not statistically significant (Fig 2B).

In summary, although cranberry alone did not always display a significant effect on the motility of the studied UPEC, in combination with propolis, all strains showed impaired swarming.

### Variability of the UPEC to form biofilm in presence of cranberry and/or propolis

**Fig 3:**
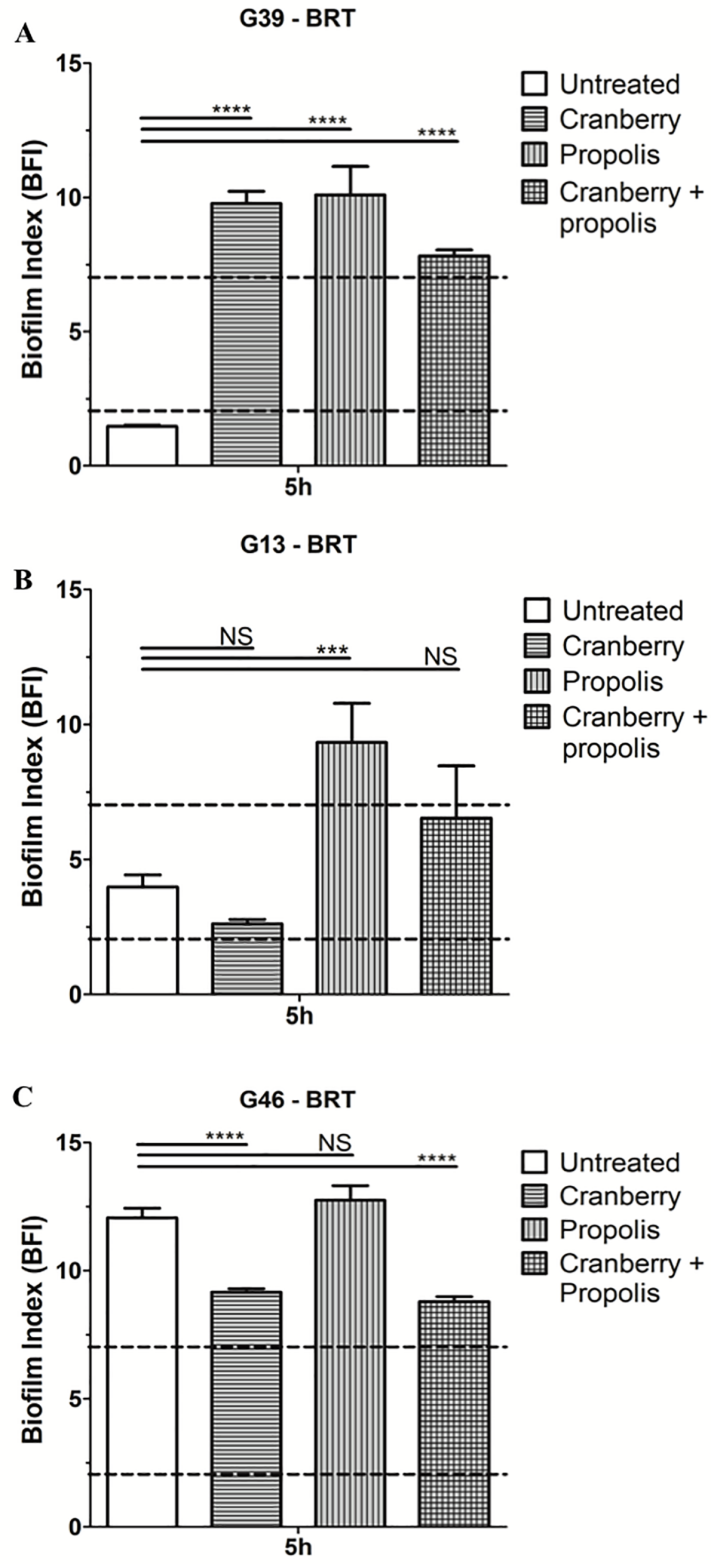
Effect of cranberry, propolis and both on early biofilm formation of a panel of uropathogenic *Escherichia coli*. The kinetics of early stages of biofilm was determined by the Biofilm Ring Test. Biofilm Index (BFI) >7 indicates an absence of biofilm with a high bacterial mobility (planktonic form) and BFI <2 indicates a fixed biofilm (sessile form). A) Profile of the G39 strain impacted by all of the treatments. B) Profile of the G13 strain impacted only by the treatment with propolis. C) Profile of the G46 strain with no biofilm formation. Means and standard errors for three independent replicate are presented. Statistical differences between different conditions at each time were obtained by ANOVA. *, p<0.01; **, p<0.001; ***, p<0.0001; NS, not significant.

The effect of cranberry ± propolis on early biofilm formation was assayed by the Biofilm Ring test (Table 2 and S1 Fig). The majority of the studied strains (7/12) showed early biofilm formation (G03, G08, G10, G19, G39, G43 and G50) (BFI < 2 after 5 hours of incubation) without treatment (Table 2). No significant effect could be noted after 2 hours (Table 2). Based on their response to cranberry ± propolis after 5 hours, strains could again be classified into three groups, independent of the virulence and resistance profiles of the isolates (Table 2). Five strains (G03, G08, G10, G19, G39) are impacted by all the treatments (except G08 with propolis) with a strong reduction of early biofilm formation (Table 2, Fig 3).

**Table 2:** Early biofilm formation (at 2 hours and 5 hours) of a panel of uropathogenic *Escherichia coli* studied without treatment, with cranberry, propolis and a combination of both.

	Biofilm ring test Mean +/-SD															
	Untreated (U)		Cranberry (C)		Propolis (P)		Cranberry + Propolis (C+P)		*p* at 2 hours				*p* at 5 hours			
Strains	2 hours	5 hours	2 hours	5 hours	2 hours	5 hours	2 hours	5 hours	U vs C		U vs P	U vs C+P	U vs C	U vs P		U vs C+P
G03	12.30 ± 0.86	5.68 ± 1.10	8.38 ± 0.19	8.85 ± 0.70	11.85 ± 1.37	11.98 ± 1.85	8.58 ± 0.83	8.33 ± 0.32	NS		NS	NS	<0.0001	<0.0001		<0.0001
G08	8.31 ± 1.00	1.57 ± 0.10	7.33 ± 0.73	8.60 ± 0.49	12.90 ± 1.73	3.85 ± 1.91	9.43 ± 1.23	8.28 ± 0.29	NS		NS	NS	<0.0001	NS		<0.0001
G10	8.58 ± 1.00	1.90 ± 0.25	7.85 ± 1.03	8.18 ± 1.36	11.83 ± 1.88	9.03 ± 1.29	9.48 ± 0.88	10.48 ± 0.62	NS		NS	NS	<0.0001	<0.0001		<0.0001
G19	9.71 ± 0.92	1.65 ± 0.10	7.43 ± 1.09	9.30 ± 1.16	11.65 ± 0.57	7.95 ± 2.91	8.18 ± 0.59	9.48 ± 0.83	NS		NS	NS	<0.0001	<0.0001		<0.0001
G39	10.01 ± 0.21	1.48 ± 0.09	9.45 ± 0.21	9.78 ± 0.92	11.00 ± 1.46	10.10 ± 2.12	10.13 ± 1.04	7.83 ± 0.45	NS		NS	NS	<0.0001	<0.0001		<0.0001
G50	2.43 ± 0.15	1.58 ± 0.05	3.68 ± 0.63	2.05 ± 0.17	5.1 ± 1.67	4.23 ± 2.89	7.08 ± 0.15	3.28 ± 0.79	NS		NS	NS	NS	0.004		NS
G13	7.62 ± 1.08	3.98 ± 1.11	7.77 ± 1.14	2.62 ± 0.41	11.70 ± 0.45	9.34 ± 1.25	8.38 ± 0.86	6.54 ± 0.33	NS		NS	NS	NS	<0.001		NS
G29	11.28 ± 0.26	4.88 ± 1.14	9.34 ± 0.73	4.50 ± 1.37	12.40 ± 1.07	9.60 ± 1.20	7.60 ± 1.20	5.42 ± 1.40	NS		NS	NS	NS	<0.0001		NS
G06	9.32 ± 0.68	9.62 ± 0.15	10.62 ± 1.08	10.90 ± 0.56	7.53 ± 1.17	11.91 ± 0.48	5.45 ± 1.31	5.73 ± 1.60	NS		NS	NS	NS	NS		NS
G24	11.27 ± 0.54	7.81 ± 3.89	8.58 ± 0.40	8.61 ± 1.75	11.70 ± 0.69	12.82 ± 0.45	8.90 ± 0.63	5.61 ± 1.44	NS		NS	NS	NS	NS		NS
G43	2.26 ± 0.29	1.50 ± 0.01	5.58 ± 1.04	1.78 ± 0.08	2.08 ± 0.56	2.15 ± 0.52	5.23 ± 0.55	1.63 ± 0.13	NS		NS	NS	NS	NS		NS
G46	12.07 ± 0.92	8.83 ± 1.12	9.17 ± 0.34	7.25 ± 0.51	12.75 ± 1.14	10.88 ± 0.95	8.81 ± 0.37	8.43 ± 0.94	NS		NS	NS	NS	NS		NS

The early biofilm was explored using the Biofilm Ring Test. The results are presented by means and standard deviation of the values of Biofilm Formation Index (BFI).

For three strains (G13, G29 and G50), an impact on the early biofilm formation has been only noted after treatment with propolis (Table 2). Finally, none of the treatments impacted early biofilm formation for four strains (G06, G24, G43 and G46). In summary, a great variability of action of cranberry and propolis was observed on early biofilm formation.

**Fig 4:**
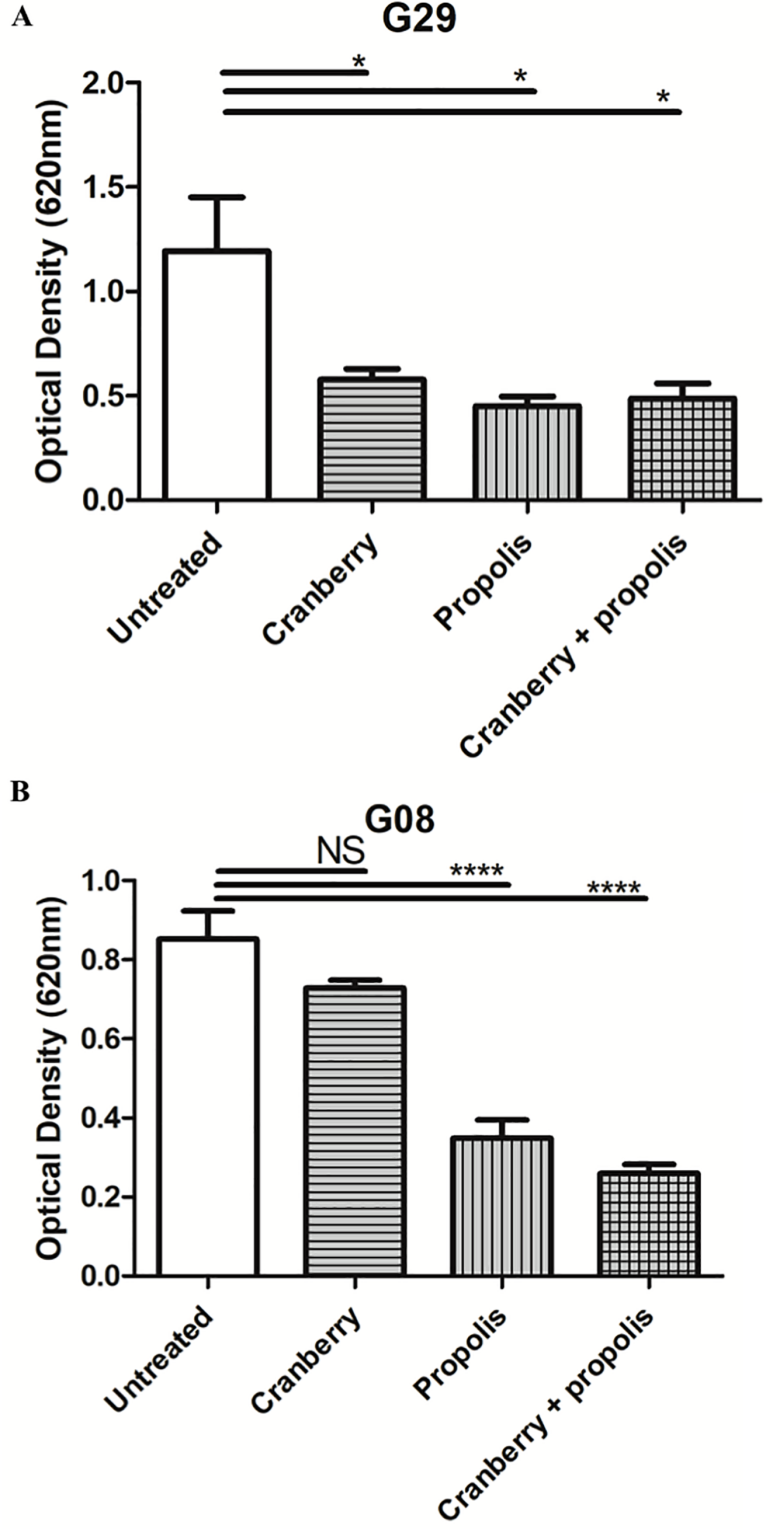
Effect of cranberry, propolis and both on complete biofilm formation of a panel of uropathogenic *Escherichia coli*. The kinetics of the complete biofilm formation were determined by crystal violet experiment. The optical density (OD) is directly linked to the biofilm formation. A) Profile of the G29 strain impacted by all the treatments. B) Profile of the G08 strain impacted only by the treatment with propolis alone and the combination of cranberry and propolis. Means and standard errors for three independent replicates are presented. Statistical differences between different conditions at each time were obtained by ANOVA.*, p<0.01; **, p<0.001; ***, p<0.0001; NS, not significant.

To evaluate whether these results extended to the formation of the complete biofilm, crystal violet assays were performed. Two profiles were observed (Fig 4). Half of the strains were significantly impacted by all the treatments compared to their standard behaviour (G03, G06, G13, G19, G29 and G43) (p<0.001) (Table 3 and S2 Fig). The other half of the studied strains was not impacted by the cranberry (G08, G10, G24, G39, G46 and G50) but was significantly affected by propolis alone and cranberry + propolis (p<0.001) (Table 3).

**Table 3:** Determination of complete biofilm formation of a panel of uropathogenic *Escherichia coli* studied without treatment, with cranberry, propolis and a combination of both.

	Crystal Violet							
	Untreated (U)	Cranberry (C)	Propolis (P)	Cranberry + Propolis (C+P)		*p*		
Strains	Mean	Mean	Mean	Mean	U vs C		U vs P	U vs C+P
G03	0.819 ± 0.232	0.396 ± 0.129	0.306 ± 0.138	0.451 ± 0.131	<0.0001		<0.0001	0.0016
G06	0.709 ± 0.310	0.372 ± 0.038	0.379 ± 0.147	0.401 ± 0.101	0.0052		0.0055	0.0084
G13	0.719 ± 0.147	0.555 ± 0.108	0.387 ± 0.161	0.332 ± 0.104	0.0052		0.0012	<0.0001
G19	0.712 ± 0.278	0.375 ± 0.036	0.425 ± 0.231	0.306 ± 0.131	0.0045		0.0087	0.0022
G29	1.194 ± 0.727	0.579 ± 0.143	0.451 ± 0.131	0.488 ± 0.204	0.0143		0.0072	0.0092
G43	1.096 ± 0.443	0.737 ± 0.124	0.450 ± 0.072	0.530 ± 0.220	0.0127		0.0028	0.006
G08	0.852 ± 0.201	0.728 ± 0.055	0.349 ± 0.130	0.259 ± 0.067	NS		0.004	<0.0001
G10	0.621 ± 0.318	0.517 ± 0.190	0.360 ± 0.125	0.329 ± 0.095	NS		0.0053	0.0026
G24	0.855 ± 0.571	0.602 ± 0.208	0.347 ± 0.172	0.479 ± 0.157	NS		<0.0001	0.0044
G39	1.282 ± 0.457	0.981 ± 0.191	0.313 ± 0.057	0.434 ± 0.147	NS		<0.0001	0.0053
G46	1.014 ± 0.440	0.762 ± 0.203	0.386 ± 0.131	0.529 ± 0.182	NS		<0.0001	0.0012
G50	1.017 ± 0.349	0.812 ± 0.185	0.281 ± 0.124	0.590 ± 0.158	NS		<0.0001	0.0071

The complete biofilm was explored using the crystal violet method. The results are presented by means and standard deviation of the values of OD620. SD, Standard Deviation; NS, not significant

## Discussion

Due to the emergence and diffusion of multidrug resistant bacteria (particularly among *E*. *coli*), new strategies to fight against these microorganisms are essential [34]. Different strategies have been developed. Some have targeted the two key metabolic pathways for a large panel of UPEC: motility and biofilm formation. They use nanostructured materials to convey antimicrobials, to transport drugs into the site of infection or to possess antimicrobial activity by themselves [34,35]. These solutions could release antimicrobial agents directly on the core of the biofilm and could have a major impact on bacterial growth [36,37]. In the same way, the use of carbohydrate-based surfactants is also a new research approach, disturbing also the bacterial motility and the biofilm formation [38–40]. The aim of this study was to evaluate the impact of two natural products: cranberry, propolis and the combination of both, on the same pathways. Some studies have already shown the impact of the cranberry and the propolis on the motility of bacteria [14,41]. Cranberry has been shown to reduce the expression of *fliC*, which encodes a major compound of the flagella, in *E*. *coli* CFT073 [14] and of *flaA*, *flhD* and *ureD* in *P*. *mirabilis* [18]. However, this effect has not yet been confirmed in UPEC. Moreover, the impact of propolis on motility was noted on *Bacillus subtilis* [42] and *Pseudomonas aeruginosa* [43]. Very recently, we described the transcriptomic impact of the combination of cranberry and propolis on the motility and the biofilm formation on a UPEC strain [44]. Thus we observed that this association had a negative impact of the gene expression related to the flagella, the fimbriae and key factors of the biofilm formation (production of exopolysaccharide, cellulose and chemoreceptors). To extend these results, it would be important to investigate the effect of this combination of cranberry and propolis on the motility and biofilm formation of a panel of UPEC.

Here, we observed the variability of the effect of cranberry ± propolis on the motility of a panel of UPEC. Swimming and swarming are complementary in the pathogenesis of UTIs; swimming is related to the liquid displacement (spread in urine) and swarming to semi-solid displacement (spread at the surface of epithelial cells) [32]. We observed: 1) a variation of the effect of cranberry alone on the mobility of the panel of the UPEC strains; 2) a strong impact of cranberry + propolis on the studied strains. Importantly, despite swimming and swarming relying on different activation factors, cranberry + propolis treatment has a clear effect on both pathways in the majority of UPEC, regardless of their virulence and resistance profiles. This combination of compounds was not efficient against the swimming of only two strains (G03 and G43), but still inhibited swarming (Figs 1C and 2B).

We also observed the same variability of the effect of cranberry ± propolis on the biofilm formation of the studied strains. Biofilm formation has serious clinical implications. Biofilm in the uroepithelium of the bladder has been suggested as the mechanism responsible for recurrent cystitis [10,45]. Moreover, CAUTIs are a serious problem during hospitalization, which is caused by a formation of a biofilm on the catheter [37]. In this study, we investigated the early formation of biofilm (<5 hours) and the complete biofilm formation (48 hours). The cranberry alone had a slight impact on the biofilm formation; only half of the studied were impacted (early and complete biofilm formation). The propolis also had a moderate effect on early biofilm formation (seven strains affected). Moreover, propolis inhibits complete biofilm formation of all the studied strains. Interestingly, although we observed a variable effect of cranberry and propolis alone on biofilm formation, the combination clearly impairs formation, indicating that the propolis could potentiate the effect of cranberry on both the early and the complete biofilm formation. These results were expected because the biofilm formation is linked to other metabolic pathways impacted by the cranberry: motility and adhesion [7,12,13,16,21,26,27,46,47]. The effects observed on motility and biofilm formation of our studied strains by cranberry + propolis suggest that the effects of these two compounds are not limited to adhesion, but have a global impact on the bacteria. A previous study has already recorded the negative impact of cranberry on complete biofilm formation on the reference *E*. *coli* strain (CFT073) [48] and *E*. *faecalis* [20].

Finally, one of the major findings in this study is the variation of effect of cranberry alone against a panel of 12 UPEC. This variation could be due to difference of baseline characteristics of these strains, the difference of clinical origins of the strains (colonisation, cystitis or pyelonephritis), or the difference of virulence and/or resistance profiles. However, no correlation was noted. The variability of the impact of the cranberry on the different UPEC may therefore be due to the presence/absence of a specific activator or regulator. This important observation could explain the variability of results observed in clinical practice with cranberry [2]. Nonetheless, the clear effect observed by the combination of propolis and cranberry suggests that this treatment represents an interesting solution to prevent UTIs.

## Conclusion

This study shows that the combination of cranberry and propolis has a strong impact on the motility and the biofilm formation of a collection of UPEC. This association could be a promising solution to prevent UTIs in the future, regardless of the virulence and resistance profiles of the UPEC.

## Supporting information

S1 Fig Of the main results obtained for the early biofilm formation (at 5 hours) of a panel of uropathogenic *Escherichia coli* studied without treatment, with cranberry, propolis and a combination of both. The early biofilm was explored using the Biofilm Ring Test. The results are presented by means and standard deviation of the values of Biofilm Formation Index (BFI). Statistical differences between untreated strains and the different conditions were obtained by ANOVA.*, p<0.01; **, p<0.001; ***, p<0.0001; NS, not significant.
(TIFF)

S2 Fig Representation of complete biofilm formation of a panel of uropathogenic *Escherichia coli* studied without treatment, with cranberry, propolis and a combination of both. The complete biofilm was explored using the crystal violet method. The results are presented by means and standard deviation of the values of OD620. Statistical differences between untreated strains and the different conditions were obtained by ANOVA.*, p<0.01; **, p<0.001; ***, p<0.0001; NS, not significant.
(TIFF)

## Abbreviations

BFI: BioFilm Index
BHI: Brain-Heart Infusion
CAUTI: catheter-associated UTI
LB: Luria Bertani
NS: Non significant
PAC-A: A-type proanthocyanidins
PBS: Phosphate Buffered Saline
SD: Standard Deviation
UTI: Urinary Tract Infections
UPEC: Uropathogenic *Escherichia coli*
