# Supplementary figures and images for: Propolis potentiates the effect of cranberry (Vaccinium macrocarpon) in reducing the motility and the biofilm formation of uropathogenic Escherichia coli
Source: PLoS One. 2018 Aug 23;13(8):e0202609. doi: 10.1371/journal.pone.0202609 (PMC6107218; doi:10.1371/journal.pone.0202609)

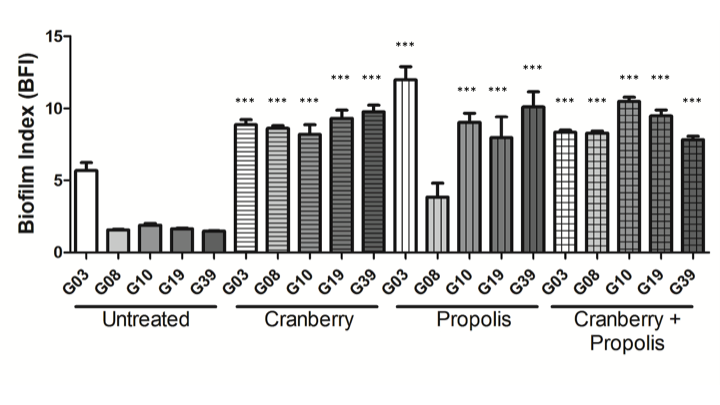

Supplement: S1 Fig — The early biofilm was explored using the Biofilm Ring Test. The results are presented by means and standard deviation of the values of Biofilm Formation Index (BFI). Statistical differences between untreated strains and the different conditions were obtained by ANOVA.*, p<0.01; **, p<0.001; ***, p<0.0001; NS, not significant. (TIFF) [file pone.0202609.s001.tiff]

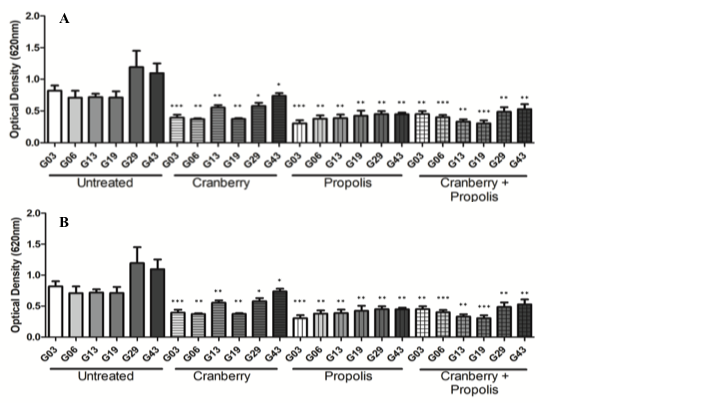

Supplement: S2 Fig — The complete biofilm was explored using the crystal violet method. The results are presented by means and standard deviation of the values of OD620. Statistical differences between untreated strains and the different conditions were obtained by ANOVA.*, p<0.01; **, p<0.001; ***, p<0.0001; NS, not significant. (TIFF) [file pone.0202609.s002.tiff]
